# Periumbilical giant abscess and intestinal leakage in late pregnancy: A rare case report and literature review

**DOI:** 10.1097/MD.0000000000034529

**Published:** 2023-08-11

**Authors:** Xiaobin Chen, Lei Xu, Zhaojun Xu, Zuyou Fan, Lie Wang, Yafeng Qi, Chen Lin

**Affiliations:** a Department of General Surgery, Fuzong Clinical Medical College, Fujian Medical University, Fuzhou, China; b Department of General Surgery, 900th Hospital of Joint Logistics Support Force, Fuzhou, China; c Graduate School, Qinghai University, Xining, China; d Department of General Surgery, Dongfang Hospital of Xiamen university, School of Medicine, Xiamen University, Fuzhou, China.

**Keywords:** after appendix surgery, intestinal fistula, periumbilical abscess, pregnancy

## Abstract

Complicated Periumbilical abscess in late pregnancy is rare in clinical practice. Pubmed searches for articles published from January 1980 to September 2021. Such related reports did not retrieve article about “pregnancy” and “periumbilical abscess.”

**Case presentation:**

We reported on a 34-year-old female patient who was admitted to the hospital with periumbilical pain for 3 days at 34 + 1 weeks of pregnancy. The result of imaging examination showed that there was an inflammatory mass in the middle and lower abdominal wall in the third trimester of pregnancy. The periumbilical abscess was punctured and drained first, and then the pregnant woman was assisted to give birth to a baby girl through vagina after the condition was stable.

Subsequently, laparotomy + abdominal abscess resection and drainage + partial small bowel resection + ileostomy were performed. Pathology showed inflammatory mass.

**Conclusions:**

Periumbilical abscess in the third trimester of pregnancy is rare clinically. For some pregnant women with previous trauma and surgical history, obstetric examination should not be restricted. For example, pregnant women with a history of abdominal surgery should expand the range of abdominal color Doppler ultrasound during the prenatal examination. When necessary, combine with computed tomography for diagnosis and treatment, avoid missed diagnosis, which will make the treatment more difficult and increase the risk. If the pregnant women has corresponding symptoms in the third trimester, vaginal delivery can be performed to terminate the pregnancy, and then the periumbilical abscess can be removed. At the same time, closely monitor the vital signs of newborn and mothers.

## 1. Introduction

Periumbilical abscess in the third trimester of pregnancy is rare clinically, and it is easy to be misdiagnosed.^[1]^ Through Pubmed, the literatures published from January 1980 to September 2021, articles about “pregnancy” and “periumbilical abscess” have not been retrieved. Here, we report a case of intestinal fistula induced by a woman in the third trimester after acute appendicitis operation. After relieving symptoms through puncture and drainage, the patient gave birth to a baby girl through vagina, and finally underwent abdominal abscess resection and drainage combined with partial resection of small intestine and terminal ileostomy. The follow-up results showed that the prognosis of the patients was good, the babies and parturients were healthy, and there were no related sequelae. We discussed the management considerations inherent in this complex clinical scenario, including clinical acuity, the time from presentation to operation management, and the importance of multi-disciplinary approach to patient management.

## 2. Case presentation

**Figure 1.**
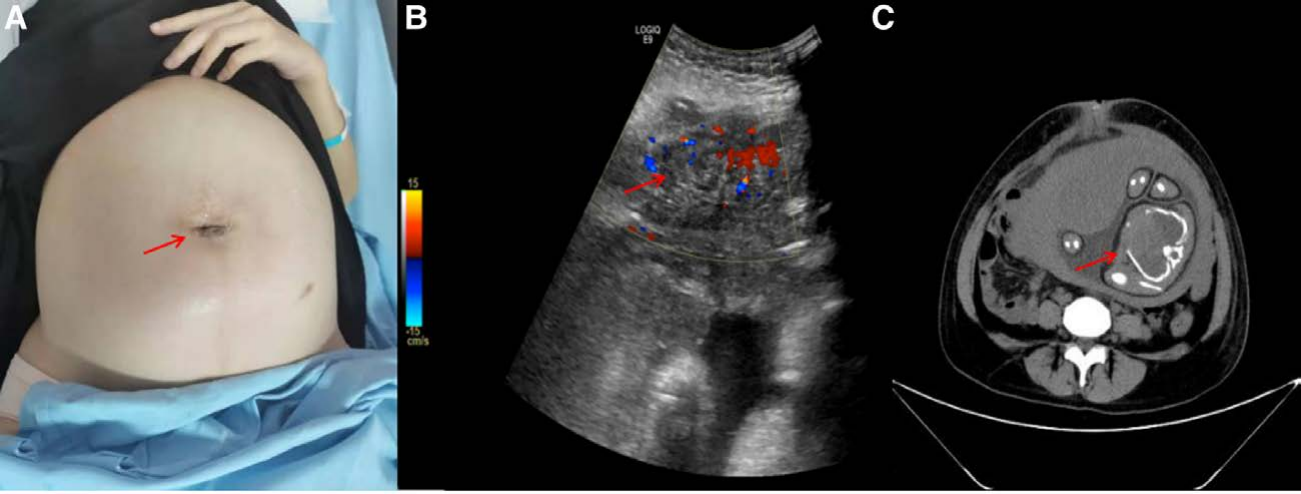
Body surface image (A) showing redness and swelling around the umbilicus with a size of about 5 cm. Color Doppler Ultrasound (B) showing the abdominal wall around the umbilicus was descending and mixed masses, with an area of about 18.4 × 43.1 cm, extending down to the suprapubic bone. Abdominal computed tomography (CT) (C) showing the uterus is enlarged, a fetus is seen in the uterine cavity, and there is no umbilical cord echo around the fetal neck; fetal position: head position, the fetal head is above the symphysis pubis. Stable fetal vital signs.

A 34-year-old woman was admitted to our hospital because of “34 + 1 weeks of pregnancy and 3 days of periumbilical pain.” Three days before the treatment, there was no obvious cause for periumbilical pain with periumbilical redness, with a range of about 3 cm. On the second day, the redness expanded to 5 cm (Fig. 1A), and the pain was not relieved. So she went to the outpatient department of our hospital for color Doppler ultrasound examination. The results showed that there was a mixed mass under the abdominal wall around the umbilicus, with a range of about 18.4 × 43.1 cm, and umbilical inflammation with inflammatory mass in the middle and lower abdominal wall was considered (Fig. 1B). For further treatment, the outpatient department was admitted with “gestational women with periumbilical abscess; fetal growth restriction in utero.” Two years ago, the patient underwent laparoscopic appendectomy after being given anti-infective symptomatic treatment in Fujian Provincial Hospital for perforation of appendix for 1 month, and there was no other operation or major trauma history; Deny the history of hepatitis and other infectious diseases.

The physical examination of the patient was crenerally normal at the time of admission. The abdomen is slightly swollen, and the scar healed by laparoscopic puncture operation can be seen. The skin around the umbilicus is red, swollen, tender, and fluctuating, and the local skin temperature is slightly higher. There is no obvious abnormality in the rest. Relevant examinations were completed after admission: blood routine: white blood cells 13.47 × 10^9^/L; blood biochemistry, coagulation function and related tumor markers were not abnormal. The results of abdominal computed tomography (CT) showed that the uterus was enlarged, a fetus was visible in the uterine cavity; and the fetal vital signs were stable (Fig. 1C).

**Figure 2.**
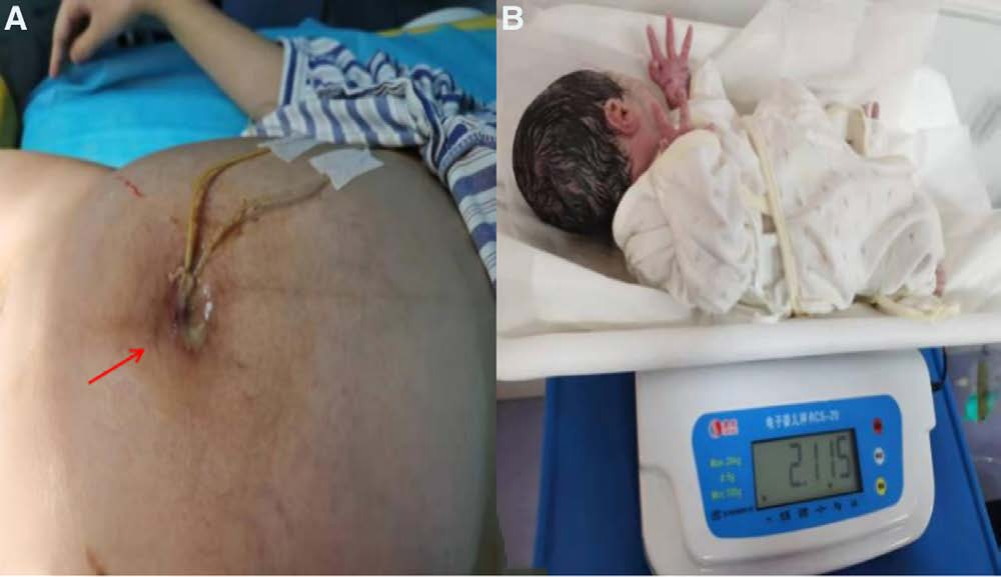
(A) Color Doppler ultrasound-guided puncture and drainage of periumbilical abscess. (B) Uterine water sac was used to induce labor, and a baby girl was delivered smoothly under the protection of perineum. The fetal position was LOA, Apgar score was 10, weight was 2115 g, length 45 cm, and appearance was not deformed. LOA = fetal position left anterior occipital.

We performed puncture and drainage of periumbilical giant abscess under the guidance of color Doppler ultrasound on the day of admission (Fig. 2A). The procedure went smoothly. After the operation, the patient self-reported abdominal pain was significantly relieved. Fetal heart monitoring indicates fetal heart rate monitoring without stimulation, through this examination to observe the changes of fetal heart rate during fetal movement, in order to understand the fetal reserve capacity reaction type, with a heart rate of 150 beats/min. After the operation, the patients were given symptomatic and supportive treatment such as “Ceftriaxone sodium + Ornidazole” anti-infection and fluid rehydration were given, and the condition did not progress during the period.

At 14:00 on the second day, the patient complained of worsening abdominal pain, and yellow drainage was visible in the abdominal negative pressure drainage tube, which was similar to intestinal contents. Yellow drainage was seen in the negative pressure drainage tube of the abdominal cavity, which was similar to intestinal contents. The possibility of pregnancy complicated with intestinal leakage was considered. Therefore, fasting and gastrointestinal decompression were given. Blood routine examination showed that white blood cells was 10.32 × 10^9^/L, and there was no obvious inflammatory reaction such as fever. After evaluating the fetal lungs maturity (we took a small amount of amniotic fluid and measured that the ratio of lecithin to sphingomyelin in the amniotic fluid. The result showed that it was >2, indicating that the fetal lung was mature), in order to ensure the safety of the fetus and the parturient, it was decided to terminate the pregnancy. In order to reduce the chance of wound infection, we usually prefer vaginal delivery, and closely check the vital signs of pregnant women and babies during delivery. At the same time, prepare for cesarean section if vaginal delivery fails. In addition, the adverse effects of drugs on the fetus should be reduced as much as possible. At 18:50, we induced labor through intrauterine water sac, and at 11:18 the next day, we successfully delivered a baby girl via vagina. Fetal position left anterior occipital, Apgar score 10 points, weight 2115 g, length 45 cm, appearance without deformity (Fig. 2B).

**Figure 3.**
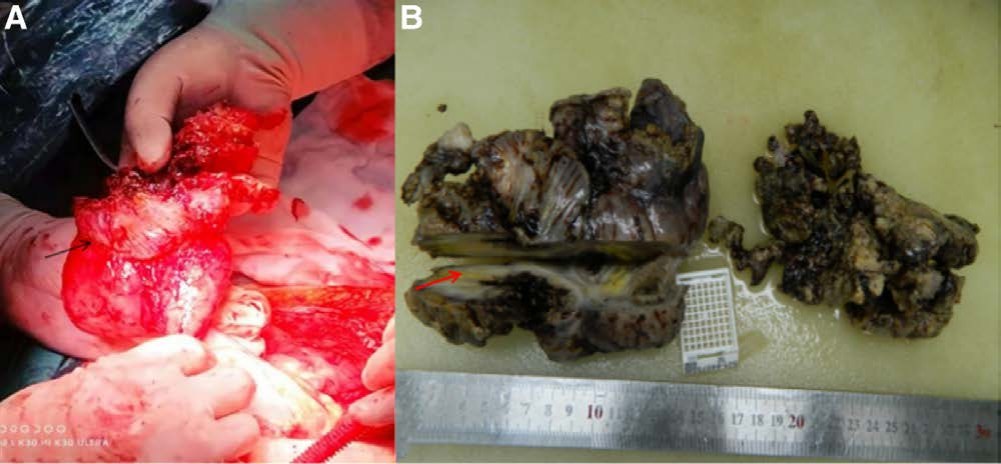
The part of the ileum about 20 cm away from the ileocecal area was adhered to the right abdominal wall, and the intestinal tube showed inflammatory edema changes, about 10 cm in length. The abdominal wall of the adhesion part of the intestine and the abdominal wall was excised separately, and the adhesion was cut. It can be seen that there is a sinus breach in the intestine and abdominal wall tissue, with a diameter of about 3 cm. (A) In the body, (B) In vitro.

**Figure 4.**
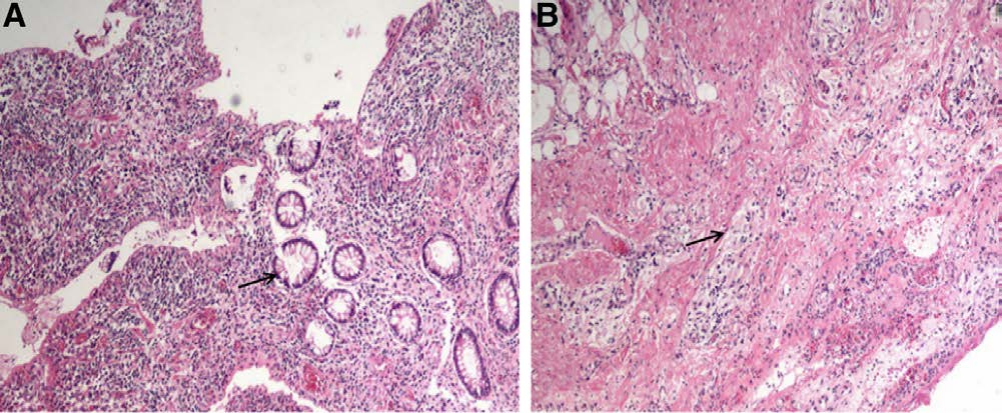
Acute and chronic inflammatory cell infiltration, vasodilatation and congestion, focal abscess formation, inflammatory exudation and necrosis were seen throughout the entire intestine; the lymph nodes detected around the intestine showed reactive hyperplasia (A, B).

**Figure 5.**
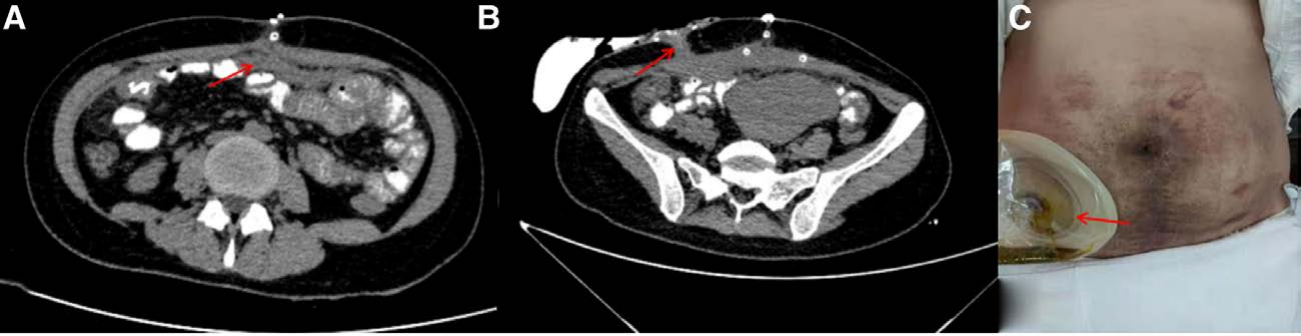
Stoma position of right lower abdomen, small intestine showed postoperative changes. (A, B) Reexamination of whole abdominal computed tomography (CT) after operation. (C) Body surface image.

At 14:13 on the third day, the second operation was performed and an exploratory laparotomy was performed under general anesthesia. During the operation, a part of the ileum about 20 cm away from the ileocecus was seen to adhere to the right abdominal wall, and the intestinal tube showed inflammatory edema changes, about 10 cm in length. After incising the adhesion, we could see a sinus tract with a diameter of about 3 cm in the bowel and abdominal wall tissue. We performed abdominal abscess excision and drainage combined with partial small bowel resection and terminal ileostomy. During the operation, the purulent tissue of abdominal wall, including the posterior sheath of rectus abdominis in the lower abdomen, with an area of about 20 × 15 cm, was removed, at the same time, the perforated intestinal tube was removed, and the terminal ileostomy was performed at the middle and outer 1-third of the line connecting the right anterior superior iliac spine and the umbilical cord (Fig. 3A and B). Postoperative pathological examination revealed acute and chronic inflammatory cell infiltration, vasodilation and congestion, focal abscess formation, inflammatory exudation, and necrosis were found in the full thickness of the bowel; the lymph nodes around the bowel showed reactive hyperplasia (Fig. 4). Regular dressing changes, pain relief, anti-inflammatory and other symptomatic treatments were given after operation. On the 14th day after operation, the results of the full-abdominal CT reexamination showed postoperative changes (Fig. 5A). On the 26th day of admission, the patient was in stable condition, recovered well after the operation, and was discharged from the hospital (Fig. 5B and C).

## 3. Discussion

Intra-abdominal abscess is a common disease in general surgery. It is more common in acute and chronic inflammation, stones and tumors in the internal organs of the abdominal cavity, as well as perforation and rupture of the digestive tract.^[2,3]^ Periappendiceal abscess caused by conservative treatment of chronic appendicitis and appendectomy is not uncommon in clinically practice, but the cases of abdominal wall abscesses following appendectomy are relatively rare, and it is even rarer in the third trimester. From January 1980 to September 2021, Pubmed searched the literature published and there were no reports of “pregnancy” and “periumbilical abscess.”

This patient was admitted to the hospital with the main complaint of “34 + 1 weeks of pregnancy, periumbilical pain for 3 days.” After abdominal doppler ultrasonography, a giant abscess in the middle and lower abdominal wall was considered. When analyzing the etiology of this patient, the author believes that it is inseparable from the following factors: The patient has a history of laparoscopic appendectomy. This abdominal abscess is considers to be caused by intestinal perforation caused by Trocar site hernia, and finally abdominal abscess caused by abdominal infection. A systematic review published by Helgstrand et al^[4]^ believes that Trocar foramen hernias most often occur in the umbilical area, and more than 80% of Trocar foramen hernias occur in Trocar sites with a diameter of ≥10 mm. In addition, there are reports in the literature that the incidence of umbilical hernia during pregnancy is 0.08%^[5]^; Peritoneal abscess of the patient due to other reasons, and intestinal fistula and periumbilical abscess were formed after invading navel and intestinal canal. infringement of the belly button, intestinal tract, intestinal fistula, periumbilical abscess; Endocrine changes in pregnant women cause tissue relaxation, vasodilatation, and increase the possibility of abscess; Pregnant women are emotionally unstable and unhealthy living habits reduce the resistance of pregnant women, which is also an important factors in inducing this disease.

When the patient was admitted to the hospital, his periumbilical symptoms were obvious and the condition was serious. So on the day of admission, the periumbilical abscess was punctured and drained. According to the related literature, drainage is an important part of surgery. Catheterization is the basic principle of abscess drainage, and it also plays a vital role in preventing the formation of complications.^[6]^ In this case, the patient symptoms aggravated after relief. The author considered abdominal wall puncture and drainage increased the possibility of intestinal perforation, because the patient was in the third trimester of pregnancy, the enlarged uterus pushed the intestines tube to the upper abdomen, and the position of the intestines changes. In addition, the patient has a previous history of appendectomy, part of intestinal tube and abdominal wall adhesion, and the necrotic tissue and secretion in the periumbilical abscess stimulate the intestinal tract to cause intestinal fistula, which is encapsulated, and the patient systemic symptoms are hidden. Therefore, the patient with intestinal fistula symptoms do not rule out the cause of puncture of the abscess puncture.^[7]^

In short, periumbilical giant abscess in the third trimester of pregnancy are relatively rare. For some pregnant women with a history of trauma and surgery, obstetric examinations should not be limited. For example, pregnant women with a history of abdominal surgery should expand the range of abdominal color Doppler ultrasound during obstetric examination. Because ultrasound imaging has the characteristics of accurately location, nature, relationship with adjacent blood vessels, low cost, and low infection rate, ultrasound-guided puncture drainage has become the most important and practical method for the clinical treatment of abdominal abscess.^[8]^ However, Yao Liqin et al^[9]^ pointed out through clinical analysis that conventional ultrasound cannot effectively show the necrotic area of abscess congestion and edema that has not yet liquefied, and the same conventional ultrasound cannot fully identify the real necrotic area inside the abdominal abscess. The reason may be that some of the abscesses are pus Fluid thickening may be weak to hypoechoic on color Doppler ultrasonography, or due to the use of antibiotics, the abscess is in a critical state that is neither liquefied nor difficult to absorb. In this case, CT should be combined for diagnosis and treatment when necessary. Although CT and other radiation examinations should be routinely avoided to cause damage to the fetus, related literature reports that the radiation measurement within 100 mGy has little effect on the fetus, and the radiation dose of abdominad CT to the fetus it is only 13 mGy^[10]^; and CT abdominal cavity can not only show the location of abscess, but also indicate the location of digestive tract perforation to a certain extent, which is helpful to guide surgical operations.^[11]^ Some studies have confirmed that conventional abdominal CT spiral scanning does not cause a significant increase in the risk of fetal radiation^[12]^; therefore, clinical medical staff can assess the risk through multi-disciplinary consultations and decide whether to combine CT for diagnosis. The author believes that patients with periumbilical abscess in late pregnancy cannot blindly undergo surgical puncture and drainage. It is recommended to perform puncture under the guidance of B-ultrasound. When B-ultrasound is difficult to diagnose, multi-disciplinary comprehensive assessment of the patient condition can be combined with CT diagnosis as appropriate. Considering the impact of CT on the fetus has not been clearly reported, it can’t be used as routine pregnancy screening.

With adequate drainage and active conservative anti-infective treatment, the infection is controlled, and the normal growth of the fetus and the life safety of pregnant woman are not affected. We can choose to continue anti-infective and conservative treatment, and pay close attention to the vital signs and changes of the patient and the fetus. Taking into account the abdominal abscess caused by appendicitis operation in pregnant women, the use of antibiotics is relatively limited, and the abscess is large, the effect of antibiotics alone is not good. If conservative treatment is used, delivery should be made as soon as possible once fetal distress in the uterus or the condition of the pregnant woman deteriorates. Related literature reports^[13]^ that when the fetus has a gestational age of ≥32 weeks, its various system functions have been fully developed, and the postpartum survival is relatively high. In this case, the pregnant woman has been pregnant for 34 weeks, and the fetal survival rate after induction of labor was high. In order to prevent the abscess from spreading during the exploration or the condition aggravates the fetal distress in the uterus, it may be consider to induce labor first, and then perform laparotomy. In order to reduce the chance of wound infection and avoid the spread of infection, we usually prefer vaginal delivery. If necessary, give fetal lung ripening. At the same time, pay attention to the adverse effects of drugs on the fetus. In addition, the patient perforation is located in the terminal ileum, and the inflammation and edema around the intestinal tract are severe. Considering that if the primary anastomosis is easy to cause anastomotic leakage, ileostomy should be performed first. If the inflammation and edema around the intestinal canal of the patient is not serious in clinical work, it can be directly anastomosed.

## 4. Conclusion

In short, for patients with severe abdominal infection during pregnancy, the cause of the infection must first be clarification, adequate drainage and etiological treatment should be carry out in time, and the delivery method should be selected according to the specific situations. Instead of blindly pursuing a single operation to solve all problems. We can adopt staged operations to reduce trauma and benefit patients. Finally, we hope that the case report can provide clinicians with a new perspective on the diagnosis and treatment of this rare disease and emergency preparation programs for such diseases.

## Acknowledgments

The authors would like to thank all colleagues for data collection from the Department of General Surgery, 900th Hospital of Joint Logistics Support Force.

## Author contributions

**Conceptualization:** Xiaobin Chen.

**Data curation:** Xiaobin Chen, Lei Xu, Zhaojun Xu, Zuyou Fan

**Formal analysis:** Xiaobin Chen, Lei Xu, Zhaojun Xu

**Funding acquisition:** Xiaobin Chen, Lei Xu, Zhaojun Xu.

**Investigation:** Xiaobin Chen, Lei Xu, Zuyou Fan.

**Methodology:** Xiaobin Chen, Lei Xu.

**Project administration:** Xiaobin Chen.

**Resources:** Xiaobin Chen, Lei Xu, Zhaojun Xu, Zuyou Fan.

**Software:** Xiaobin Chen, Lei Xu, Zhaojun Xu, Zuyou Fan, Yafeng Qi, Chen Lin.

**Supervision:** Lie Wang, Yafeng Qi, Chen Lin.

**Validation:** Lie Wang, Yafeng Qi, Chen Lin.

**Visualization:** Lie Wang, Yafeng Qi, Chen Lin.

**Writing – original draft:** Xiaobin Chen.

**Writing – review & editing:** Lie Wang, Yafeng Qi, Chen Lin.
